# Development of Common Data Elements for Organ Transplantation

**DOI:** 10.1001/jamanetworkopen.2025.7704

**Published:** 2025-04-28

**Authors:** Lisa M. McElroy, Ursula Rogers, Liz Nichols, Nrupen A. Bhavsar, Tyler Schappe, Jessica Harding, Alexandra T. Strauss, Elisa J. Gordon, Katie Ross-Driscoll, Rhiannon Deirhoi-Reed, Norine W. Chan, Jackie B. Henson, Yue Harn Ng, Dave J. Taber, Adam J. Milam, Jesse D. Schold, Juan Carlos Caicedo, Andrew B. Adams, Rachel E. Patzer, Samuel I. Berchuck, Roland Matsouaka, Jennifer Gagnon, Allan D. Kirk

**Affiliations:** 1 Department of Surgery, Duke University School of Medicine, Durham, North Carolina; 2 Department of Population Health Sciences, Duke University School of Medicine, Durham, North Carolina; 3 Department of Biostatistics and Bioinformatics, Duke University School of Medicine, Durham, North Carolina; 4 Department of Surgery, Emory University School of Medicine, Atlanta, Georgia; 5 Department of Medicine, Johns Hopkins University, Baltimore, Maryland; 6 Department of Surgery, Vanderbilt University Medical Center, Nashville, Tennessee; 7 Center for Health Services Research, Regenstrief Institute, University of Indiana, Indianapolis; 8 Department of Surgery, University of Alabama, Birmingham; 9 Department of Medicine, Duke University School of Medicine, Durham, North Carolina; 10 Department of Medicine, University of Washington, Seattle; 11 Department of Surgery, Medical University of South Carolina, Charleston; 12 Department of Anesthesiology and Perioperative Medicine, Mayo Clinic, Phoenix, Arizona; 13 Departments of Surgery and Epidemiology, University of Colorado Anschutz Medical Campus, Aurora; 14 Department of Surgery, Northwestern University, Chicago, Illinois; 15 Department of Surgery, University of Minnesota, Minneapolis

## Abstract

This cohort study examines the validity of an electronic health record data model for organ transplantation.

## Introduction

Organ transplantation research and health policy are largely based on registry data that lack granularity, and recent federal initiatives have called for modernization of the transplant data collection process.^1^ Common data models (CDM) are standardized metadata language that allow users to share data and their meaning across applications.^2^ The Consortium for the Holistic Assessment of Risk in Transplant (CHART) developed a CDM to allow data collection that begins at transplant referral for research and quality improvement.^3^ The purpose of this cohort study is to demonstrate the validity of the CDM.

## Methods

The initial variable list was identified based on 26 semistructured interviews with multidisciplinary transplant stakeholders and group discussion at CHART steering committee meetings, then used to establish 368 data elements (eAppendix in Supplement 1). The CDM is organized into clinical domain tables with equivalence mapping variables for crosswalking. Data deidentification included randomized patient identifiers and identifiers to enable linking across tables (Figure). The unit of observation is transplant referral, and phases are defined by time from referral. A shift-and-truncate method preserves temporal relations while adhering to privacy standards.^4^ Area-level social determinants of health (SDOH) were derived by linking patient addresses to a geocoding program written by the coordinating center.^5^

**Figure.**
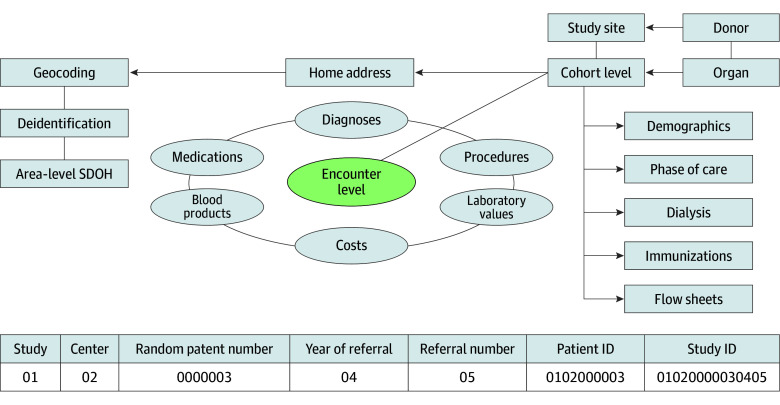
Consortium for the Holistic Assessment of Risk in Transplant Common Data Model Organization Individual components of patient and study identification numbers. ID indicates identification.

The initial extraction included adult patients referred for liver, kidney, or pancreas transplants between January 1, 2016, and December 31, 2022; data were collected from 1 year prior to referral through death, loss to follow-up, or administrative censor date. Approval was obtained from Duke University and each collaborating center’s institutional review board prior to data extraction. Extracted data were transmitted securely using a secure file transplant protocol to a Federal Information Security Modernization Act–compliant storage system. Informed consent was not required because no patients were contacted as part of this study and data were deidentified by participating centers prior to transfer to the data coordinating center (Duke University). Descriptive statistics were used to assess data quality for select variables.^6^ All analyses were performed using R version 4.3.3 (R Project for Statistical Computing). Data were analyzed from August 2023 to October 2024. This cohort study follows the Strengthening the Reporting of Observational Studies in Epidemiology (STROBE) reporting guideline. 

## Results

The cohort includes 91 877 referrals representing 79 787 patients referred for abdominal transplant (mean [SD] age, 54.3 [13.0] years; 32 473 female [40.7%]; 2.9% were Asian, 43.3% were Black, and 43.0% were White) at 7 transplant centers and includes 9 128 093 care encounters, 45 292 686 laboratory values, and 50 972 302 procedures. Missingness for cohort and demographic data elements ranged from 0 to 100%, with high missingness (ie, more than 40%) for other individual SDOH, such as education level and citizenship (Table). Missingness for the transplant phases ranged from 0.4% for transplant to 26.6% for committee review. For cohort and demographic variables, the proportion of values that were implausible ranged from 0% to 0.2%. Discordance of transplant phase temporal order ranged from 2.1% to 6.4% after excluding implausible values.

**Table.** Data Quality of Select Variables, 2016-2022

Variable	No. (%)			
	Completeness, referrals with missing values	Plausibility, values out of range	Concordance, values with discordance	Currency, referrals without a value at committee review
Cohort (n = 91 877)				
Age at referral	0	0	NA	NA
Organ type	0	0	NA	NA
Referral year	0	0	NA	NA
Demographics				
Sex	92 (0.1)	0	NA	NA
Gender identity	77 636 (84.5)	0	NA	NA
Sexual orientation	81 035 (88.2)	0	NA	NA
Ethnicity	8453 (9.2)	0	NA	NA
First patient race	4043 (4.4)	184 (0.20)	NA	NA
Second patient race	91 050 (99.1)	0	NA	NA
Highest education	70 194 (76.4)	0	NA	NA
Employment status	32 800 (35.7)	0	NA	NA
Primary language	4043 (4.4)	0	NA	NA
Marital status	1194 (1.3)	0	NA	NA
Citizenship	10 177 (51.2)	0	NA	NA
Transplant phases (n = 91 877)				
Days from referral to evaluation	8453 (9.2)	230 (0.25)	3069 (3.34)	NA
Days from referral to committee review	24 439 (26.6)	202 (0.22)	4153 (4.52)	NA
Days from referral to waitlist	2205 (2.4)	5375 (5.85)	5908 (6.43)	NA
Days from referral to transplant	368 (0.4)	92 (0.10)	2499 (2.72)	NA
Days from referral to admission for transplant	7810 (8.5)	NA	2251 (2.45)	NA
Days from referral to discharge from transplant	17 732 (19.3)	37 (0.04)	2793 (3.04)	NA
Days from referral to referral closed (resolved)	4778 (5.2)	110 (0.12)	2811 (3.06)	NA
Days from referral to death	23 245 (25.3)	184 (0.20)	1883 (2.05)	NA
Select laboratory values				
Body mass index, %	35.51	0.07	Any cause, 7.59	90 d, 79.68
Height	35.56	0.19	1.07	Any time prior, 73.67
Weight	35.12	0.49	0.93	90 d, 79.16
HbA_1c_	64.48	0.94	NA	90 d, 92.00
eGFR	47.71	1.28	NA	90 d, 83.97
Encounters, missing values, (n = 9 128 093)				
Encounter type	0	NA	NA	NA
Reason for visit	7 262 311 (79.56)	NA	NA	NA
Encounter status	6 354 978 (69.62)	NA	NA	NA
Phase history, missing values (n = 9 128 093)				
Status reason	3 595 555 (39.39)	NA	NA	NA

Abbreviations: eGFR, estimated glomular filtration rate; NA, quality metric not applicable to the variable format or definition.

## Discussion

Our findings suggest the feasibility of electronic health record (EHR)–based data assimilation from transplant referral through posttransplant outcomes, including a large collection of SDOH data not currently captured in existing national systems. We found high rates of plausibility and concordance but a wide range of missingness by data element; this reflects the known center-level variation in data collection practices, even among high-volume transplant centers. Missingness was generally clustered by EHR platform and center and decreased as patients progressed through the transplant care continuum. A unified, system-agnostic EHR-based CDM may be a superior alternative to the current registry-based national data collection systems and reduce the significant administrative burden associated with current data collection practices. Our study is limited by validation limited to liver, kidney, and pancreas patients and reports of quality metrics following iterative feedback and consultation with the coordinating center bioinformaticians. Further validation is required to determine whether a national EHR-based CDM for transplant data collection is plausible. We believe this approach should be carefully assessed in collaboration with bioinformaticians and private vendors as national regulatory bodies consider the best data reporting system for US transplant centers in the future.

## Notes

Supplement 1. **eAppendix.** CDM data variable list

Supplement 2.
Data Sharing Statement

## References

[1] Health Resources and Services Administration. HRSA announces organ procurement and transplantation network modernization initiative. May 2023. Accessed June 30, 2024. https://www.hrsa.gov/optn-modernization/march-2023

[2] Cho S, Mohan S, Husain SA, Natarajan K. Expanding transplant outcomes research opportunities through the use of a common data model. Am J Transplant. 2018;18(6):1321-1327. doi:10.1111/ajt.14892 29687963 PMC6070138

[3] McElroy LM, Reed RD, Gordon E, . CHART: harmonizing data for research, transparency and equity. Ann Surg. 2025;281(3):373-375. doi:10.1097/SLA.0000000000006410 38899463 PMC11659506

[4] Hripcsak G, Mirhaji P, Low AF, Malin BA. Preserving temporal relations in clinical data while maintaining privacy. J Am Med Inform Assoc. 2016;23(6):1040-1045. doi:10.1093/jamia/ocw001 27013522 PMC5070517

[5] Schappe T, McElroy LM, Ogundolie M, Matsouaka R, Rogers U, Bhavsar NA. A data pipeline for secure extraction and sharing of social determinants of health. PLoS One. 2025;20(1):e0317215. doi:10.1371/journal.pone.0317215 39888883 PMC11785280

[6] Lewis AE, Weiskopf N, Abrams ZB, . Electronic health record data quality assessment and tools: a systematic review. J Am Med Inform Assoc. 2023;30(10):1730-1740. doi:10.1093/jamia/ocad120 37390812 PMC10531113

