# Supplementary material for: Development of Common Data Elements for Organ Transplantation
Source: JAMA Netw Open. 2025 Apr 28;8(4):e257704. doi: 10.1001/jamanetworkopen.2025.7704 (PMC12038510; doi:10.1001/jamanetworkopen.2025.7704)
Supplement: Supplement 1. — eAppendix. CDM data variable list [file jamanetwopen-e257704-s001.pdf]

Supplemental Online Content

McElroy LM, Rogers U, Nichols L, et al. Development of common data elements for organ transplantation. *JAMA Netw Open*. 2025;8(4):e257704. doi:10.1001/jamanetworkopen.2025.7704

eAppendix. CDM Data Variable List

This supplemental material has been provided by the authors to give readers additional information about their work.

© 2025 McElroy LM et al. *JAMA Network Open*.

**eAppendix.** CDM Data Variable List		
**Variable**		**Description**
**SITE XREF**		Study key held by sites locally in a secure location; it contains dates used to calculate values in other domains and can be used to re-identify patients locally.
	Study ID	Deidentified ID representing one referral, 13 digits (e.g. 101000012301)
	Patient ID	Deidentified ID representing one patient, first 11 digits of Study ID (e.g. 1010000123)
	Episode ID	Transplant Episode ID for the patient
	Referral Number	Referral number, last two digits of the Study ID (e.g. 01)
	PX ID	Unique identifier for patient in UNOS
	PAT ID	Internal patient identifier for the Epic clarity database
	PAT MRN ID	Patient medical record number
	Patient First Name	First name of patient
	Patient Last Name	Last name of patient
	Referral Date	Date of referral (e.g. 10/31/2019)
	Year Prior Referral Date	Date one year prior to referral (e.g. 10/31/2018)
	Admin Censor Date	Randomly assigned censor date in 2022
	Last Run DTTM	Timestamp of dataset creation
**Cohort**		List of all adult abdominal transplant referrals
	Age at Referral	Patient's age at time of referral
	Episode Status	Status of referral at time of admin censor date (e.g. Active, Resolved)
	Current Stage	Current phase of referral at time of admin censor date (e.g. Referral, Evaluation, Waitlist, Transplant)
	Current Reason	Reason referral is in the current phase at the time of admin censor date (e.g. Scheduled for evaluation)
	Transplant DCD	Y/N flag indicating if a patient will accept an organ from a donor who died from cardiac death
	Organ Type	Organ(s) associated with referral
	Organ Status	Status of transplanted organ at time of admin censor date (e.g. Transplanted, Failed)
	Referral Year	Year this referral was created, 2016-2022 inclusive
	Primary Organ Disease Diagnosis	Patient's primary reason for needing a transplant
	Txp Admission Encounter ID	Encounter ID associated with the transplant surgery
	Days from Referral to Evaluation	Time from referral (T0) to evaluation state date
	Days from Referral to Committee Review	Time from Referral (T0) to committee review decision date

© 2025 McElroy LM et al. *JAMA Network Open*.

	Days from Referral to Waitlist	Time from Referral (T0) to waitlist start date
	Days from Referral to Transplant	Time from Referral (T0) to transplant surgery date
	Days from Referral to Admission	Time from Referral (T0) to hospital admission date associated with the transplant
	Days from Referral to Discharge	Time from Referral (T0) to the hospital discharge date associated with the transplant
	Days from Referral to Resolved	Time from Referral (T0) to the date the referral is closed
	Days from Referral to Censor	Time from Referral (T0) to admin censor date
	Number Referrals to Waitlist	Number of referrals for this organ before the patient is waitlisted. 0 (never waitlisted), 1 (waitlisted on first referral), 2 (first referral denied, second waitlisted)
	Prior Organ Transplant	Indicator of if a patient ever had a prior transplant of any organ type
	Previous Abdominal Surgery Waitlist	Indicator of if a patient ever had a prior abdominal transplant prior to waitlist date
	Previous Abdominal Surgery Transplant	Indicator of if a patient ever had a prior abdominal transplant prior to transplant date
	Kidney Allocation System	Policy: Kidney Allocation System major policy flag based on listing date
	Acuity Circles	Policy: Acuity Circles liver distribution policy flag based on listing date
**Demographic**		Baseline demographic information
	Study ID	Deidentified ID representing one referral, 13 digits
	Patient ID	Deidentified ID representing one patient, first 11 digits of Study ID
	Days from Referral to Death	Time from Referral (T0) to death date, if applicable
	Sex	Patient's legal sex
	Gender Identity	Patient's gender with which they identify
	Sexual Orientation	Patient's sexual orientation
	Ethnicity	The name of the ethnic group of the patient
	First Patient Race	The name of the patient race (first race listed)
	Second Patient Race	The name of the patient race (second race listed)
	Third Patient Race	The name of the patient race (third race listed)
	Highest Education	Patient's highest level of education
	Citizenship	Patient's citizenship status
	Employment Status	Patient's highest level of education
	Primary Language	Patient's preferred language to receive care
	Marital Status	Patient's marital status
**Transplant Phase History**		Progression of referrals through the transplant process
	Study ID	Deidentified ID representing one referral, 13 digits

© 2025 McElroy LM et al. *JAMA Network Open*.

	Patient ID	Deidentified ID representing one patient, first 11 digits of the Study ID
	Line	Each line represents a change in status for this referral, in chronological order (e.g. 1,2,3)
	Days from Referral to Transplant Phase	Days from T0 (Referral) to phase history date
	Stage	The phase of the referral
	Status	Status associated with this referral's transplant phase and reason
	Status Reason	Reason for this referral's transplant phase and status
**Encounters**		All encounters beginning one year prior to referral
	Study ID	Deidentified ID representing one referral, 13 digits
	Patient ID	Deidentified ID representing one patient, first 11 digits of the Study ID
	Encounter ID	Deidentified ID representing a unique patient contact
	Linked Encounter	Yes if this encounter is linked to the transplant referral
	Encounter Type	Type of encounter (e.g. Office Visit, Telephone, Video)
	Days from Referral to Encounter	Days from Referral (T0) to encounter date
	Reason for Visit	The reason for visit for the encounter (e.g. Follow-Up, Sick, Nutrition Counseling)
	Encounter Status	The status of the patient encounter (e.g. Completed, Cancelled, No Show)
	BMI	BMI associated with the encounter
	Height	Height associated with the encounter
	Weight	Weight associated with the encounter
	Heart Rate	Heart Rate associated with the encounter
	Blood Pressure	Blood Pressure associated with the encounter
	Payor Name	The primary payor for patient at time of encounter (e.g. Blue Cross, Aetna, Cigna)
	Financial Class	The financial class associated with the patient at time of encounter (e.g. Commercial, Medicare, Self-Pay)
	Tobacco Use	Description associated with the patient's Smoking Tobacco Use response (e.g. Never, Former, Some Days)
	Days from Referral to Hospital Admission	Days from referral date to the hospital admission date associated with the encounter
	Days from Referral to Hospital Discharge	Days from referral date to the hospital discharge date associated with the encounter
	Hospital Length of Stay	Total Hospital Length of Stay in days
	Transplant ICU Length of Stay	Total ICU Length of Stay from Transplant Surgery end to initial ICU departure in days

© 2025 McElroy LM et al. *JAMA Network Open*.

	Total ICU Length of Stay	Total ICU Length of Stay in Days
	Hospital Admission Type	Type of admission for this encounter (e.g. Emergency, Routine Elective)
	Hospital Admission Source	The admission source for this patient contact (e.g. Transfer, Clinic, Home)
	Discharge Disposition	Discharge disposition location (e.g. Home, Rehab, skilled nursing)
	Hospital Service	The hospital service name for this patient contact (e.g. General Surgery, Transplant, Cardiology)
	IP Final DRG	The final coded DRG stored in this admission's hospital account
	ED Disposition	The disposition of the patient when discharged from the ED (e.g. Admitted, etc.)
	Department Specialty	The specialty of the department for this visit (e.g. Transplant, Physical Therapy)
	Visit Provider Type	Type of provider this patient visited with (e.g. Physician, Nurse Practitioner)
	Visit Type	Type of visit this is (e.g. office visit)
	Patient Class	e.g. Inpatient, Outpatient, ED
**Diagnosis**		All patient diagnoses beginning one year prior to referral
	Study ID	Deidentified ID representing one referral, 13 digits
	Patient ID	Deidentified ID representing one patient, first 11 digits of the Study ID
	Encounter ID	Deidentified ID representing a unique patient contact
	Linked Encounter	Y if this encounter is linked to the transplant referral
	Diagnosis Source	The type of diagnosis, clinical and billed diagnoses
	Days from Referral to Diagnosis	Days from Referral to diagnosis date
	Diagnosis Name	ICD10 Name
	Diagnosis Group Name	The name of the diagnosis group to which the diagnosis belongs
	Current ICD9 List	ICD9 Code, if applicable
	Current ICD10 List	ICD10 Code
	Rejection Diagnosis	Flag indicating if the diagnosis refers to a transplant rejection
**Lab Results**		All patient lab results beginning one year prior to referral
	Study ID	Deidentified ID representing one referral, 13 digits
	Patient ID	Deidentified ID representing one patient, first 11 digits of the Study ID
	Encounter ID	Deidentified ID representing a unique patient contact
	Linked Encounter	Y if this encounter is linked to the transplant referral
	Order Procedure ID	Deidentified ID representing a unique order
	Procedure Code	The generic string that identifies the lab or lab panel ordered, associates with the Order Procedure ID
	Procedure Name	The name of the lab or lab panel ordered

© 2025 McElroy LM et al. *JAMA Network Open*.

	Days from Referral to Lab Order	Days from referral to order date
	Days from Referral to Specimen	Days from referral to specimen collection date
	Order Status	Status of the order (e.g. Completed)
	Component Name	Name of the lab component (e.g. Hemoglobin)
	Component Common Name	Common name of the lab component (e.g. Hemoglobin)
	Days from Referral to Result	Days from referral to result date
	Result Status	Status of the result (e.g. Final, Corrected)
	Result Value	Result value for this lab, can be non-numeric
	Reference Unit	Units for each result component value
	Reference Low Value	Lowest acceptable value for each result component
	Reference High Value	Highest acceptable value for each result component
**Ordered Medications**		All patient medications ordered beginning one year prior to referral
	Study ID	Deidentified ID representing one referral, 13 digits
	Patient ID	Deidentified ID representing one patient, first 11 digits of the Study ID
	Encounter ID	Deidentified ID representing a unique patient contact
	Linked Encounter	Y if this encounter is linked to the transplant referral
	Order Medication ID	Deidentified ID representing a unique order for the patient on this date
	Days from Referral to Order Start	Days from referral to the date the medication is supposed to start
	Days from Referral to Order End	Days from referral to the date the medication is supposed to end
	Days from Referral to Order	Days from referral to the date the order is placed
	Medication Simple Generic	The first generic name listed for each medication.
	Order Status	Current status of an order (e.g. Completed, Dispensed, Discontinued)
	Order Class	This value defines how clinical systems process the order (e.g. Normal, Historical, Phone-in)
	Ordering Mode	The ordering mode of the order (which setting this order was placed) (e.g. Inpatient, Outpatient)
	Account Baseclass	Category value corresponding to the Admission/Discharge/Transfer (ADT) patient classification for this patient contact (e.g. Inpatient, Outpatient, Emergency)
	Pharmacy Class	Pharmaceutical class indicating the chemical families the drug belongs to (e.g. Immunosuppressive)
	Pharmacy Subclass	The first pharmaceutical subclass listed for each medication (e.g. Immunosuppressive - Calcineurin Inhibitors)

© 2025 McElroy LM et al. *JAMA Network Open*.

	DEA Class Code	DEA Controlled Substance Code, which indicates this drug's abuse and dependency potentials
	Route	Route of administration of a medication (e.g. Oral, Topical)
	Discrete Dose	Discrete dose for a medication as entered by the user in the orders activity
	Dose Unit	Dosage unit of a medication
	Frequency	Discrete frequency record associated with this medication order.
	Strength	Strength of this NDC version of the drug.
	Form	Form of the medication (e.g. Tablet, Capsule)
	Quantity	Quantity of the prescription being dispensed
	Refills	Number of refills allowed for this prescription.
	Controlled Medication	Y if the DEA has designated this medication as a controlled substance; otherwise N
	Investigational Medication	Y if this medication is considered to be investigational;
	Pending Approval Flag	Pending medication approval status category for the order (e.g. Approved, Refused)
**Ordered Procedures**		All patient procedures ordered beginning one year prior to referral
	Study ID	Deidentified ID representing one referral, 13 digits
	Patient ID	Deidentified ID representing one patient, first 11 digits of the Study ID
	Encounter ID	Deidentified ID representing a unique patient contact
	Linked Encounter	Y if this encounter is linked to the transplant referral
	Order Procedure ID	Deidentified ID representing a unique order
	Days from Referral to Order	Number of days from referral to procedure
	Order Status	The status of the order (e.g. Sent, Completed)
	Order Type	The Type of order (e.g. Imaging, Lab, Occupational therapy)
	Procedure Code	The generic string that identifies the procedure, associates with the Order Procedure ID
	CPT Code	The CPT procedure code
	Procedure Name	The name of the procedure, associates with the Order Procedure ID and Procedure Code
**Billed Procedures**		All patient procedures completed beginning one year prior to referral
	Study ID	Deidentified ID representing one referral, 13 digits
	Patient ID	Deidentified ID representing one patient, first 11 digits of the Study ID
	Encounter ID	Deidentified ID representing a unique patient contact
	Linked Encounter	Y if this encounter is linked to the transplant referral, N if the encounter is not linked
	Days from Referral to Procedure	Number of days from referral to procedure completion

© 2025 McElroy LM et al. *JAMA Network Open*.

	Procedure Code	The generic string that identifies the procedure, associates with the Order Procedure ID
	Procedure Name	The name of the procedure, associates with the Order Procedure ID and Procedure Code
**Dialysis**		All patient dialysis information beginning one year prior to referral
	Study ID	Deidentified ID representing one referral, 13 digits
	Patient ID	Deidentified ID representing one patient, first 11 digits of the Study ID
	Nephrology Episode Number	Patients may have multiple nephrology episodes dependent upon their schedule and facilities, this is a unique row number associated with each episode
	Dialysis Timeframe	Indicates if the dialysis occurred pre or post transplant
	Days from Referral to Modality Start	Number of days from Referral to Modality Start Date (a dialysis patient's start date of their current treatment details)
	Days from Referral to Dialysis Start	Number of days from Referral to Dialysis Start Date (start date of a patient's dialysis treatment episode)
	Days from Referral to Dialysis End	Number of days from Referral to Dialysis End Date (end date of a patient's dialysis treatment episode)
	Dialysis Type	Patient's type of dialysis treatment
	Dialysis Episode Purpose	Purpose of the dialysis episode
**MELD History**		All patient MELD scores beginning one year prior to referral
	Study ID	Deidentified ID representing one referral, 13 digits
	Patient ID	Deidentified ID representing one patient, first 11 digits of the Study ID
	Days from Referral to MELD	Number of days from Referral to MELD Score date
	Line	Line number associated with the MELD Score
	MELD Exception Diagnosis	MELD Exception Diagnosis
	MELD Score	MELD Exception Score
**Donor**		Donor Information, all transplants
	Study ID	Deidentified ID representing one referral, 13 digits
	Patient ID	Deidentified ID representing one patient, first 11 digits of the Study ID
	Organ ID	Deidentified ID consisting of the Study ID + the organ number
	Organ Type	Organ name(s) associated with the transplant episode (referral)
	Donor Type	Specifies organ donor type
	Days from Referral to Recovery	Number of days from Referral to Recovery date
	Donor Age	Organ donor's age at death
	Height	Organ donor's height in inches
	Weight	Organ donor's weight in ounces

© 2025 McElroy LM et al. *JAMA Network Open*.

	HCV	Is donor HCV positive
	HCV NAT	Is donor HCV NAT positive
	HCV AB	Is donor HCV Ab positive
	CMV Total	Is donor CMV positive
	CMV IGG	Is donor CMV positive
	CMV IGM	Is donor CMV positive
	HIV NAT	Is donor HIV positive
	HIV AGAB	Is donor HIV positive
	HIV	Is donor HIV positive
	HBC Total	Is donor HBC positive
	EBV IGG	Is donor EBV positive
	EBV IGM	Is donor EBV positive
	Transferred on a Pump	Was the organ transported on a pump
	Clamp TM Zone	Time zone of the location where cross clamp occurred
	Days from Referral to Clamp	Number of days from Referral to Clamp date
	Clamp Time	Cross-clamp time
	Donor HLA A1	Donor HLA Phenotype
	Donor HLA A2	Donor HLA Phenotype
	Donor HLA B1	Donor HLA Phenotype
	Donor HLA B2	Donor HLA Phenotype
	Donor HLA BW4	Donor HLA Phenotype
	Donor HLA BW6	Donor HLA Phenotype
	Donor HLA C1	Donor HLA Phenotype
	Donor HLA C2	Donor HLA Phenotype
	Donor HLA DPA1	Donor HLA Phenotype
	Donor HLA DPA2	Donor HLA Phenotype
	Donor HLA DPB1	Donor HLA Phenotype
	Donor HLA DPB2	Donor HLA Phenotype
	Donor HLA DQA1	Donor HLA Phenotype
	Donor HLA DQA2	Donor HLA Phenotype
	Donor HLA DQB1	Donor HLA Phenotype
	Donor HLA DQB2	Donor HLA Phenotype
	Donor HLA DR1	Donor HLA Phenotype
	Donor HLA DR2	Donor HLA Phenotype
	Donor HLA DR51 1	Donor HLA Phenotype
	Donor HLA DR51 2	Donor HLA Phenotype
	Donor HLA DR52 1	Donor HLA Phenotype

© 2025 McElroy LM et al. *JAMA Network Open*.

	Donor HLA DR52 2	Donor HLA Phenotype
	Donor HLA DR53 1	Donor HLA Phenotype
	Donor HLA DR53 2	Donor HLA Phenotype
**Organ**		Perioperative transplant information
	Study ID	Deidentified ID representing one referral, 13 digits
	Patient ID	Deidentified ID representing one patient, first 11 digits of the Study ID
	Organ ID	Deidentified ID consisting of the Study ID + the organ number
	Organ Type	Organ name(s) associated with the transplant episode (referral)
	Out of Ice Time	Time the organ was removed from ice or pump, whichever was later
	Reperfusion Time	Perfusion time of organ minutes component
	Cardiopulmonary Bypass Time	Number of minutes the patient was on cardiopulmonary bypass for circulatory support during the surgery
	Cold Ischemic Time	Time in cold ischemia minutes
	Warm Ischemic Time	Time in warm ischemia minutes
	Total Ischemic Time	Total ischemia time in minutes
	Portal Clamp Off Time	Portal clamp off time for the transplant
	Arterial Clamp Off Time	Instant clamp taken off
	Recipient Anastomosis Time	Anastomosis start time for the transplant
	Estimated Blood Loss	Estimated blood loss for the organ recipient in milliliters
	Preservation Solution	Preservation solution used during transplant
	Crystalloid ML Admin	Amount of the crystalloid fluid administered during the transplant
	Colloid ML Admin	Amount of the colloid fluid administered during the transplant
	RBC Units Admin	Amount of the RBC fluid administered during the transplant
	FFP Units Admin	Amount of the FFP fluid administered during the transplant
	Cryoprecipitate Units Admin	Amount of the cryoprecipitate fluid administered during the transplant
	Platelets Unit Admin	Amount of platelets administered during the transplant
	Autologous PRBC Admin	Amount of autologous RBCs administered during the transplant
	Cell Saver Admin	Amount of cell saver fluid administered during the transplant
	Albumin Admin	Amount of albumin fluid administered during the transplant
	RBC ML Admin	Amount of RBC fluid administered during the transplant
	B Cells Crossmatch	Crossmatch test result
	T Cells Crossmatch	Crossmatch test result
	Days from Referral to Organ Fail	Days from Referral to the Transplant Fail Date
	Organ Fail Determined By	Method by which organ failure was determined

© 2025 McElroy LM et al. *JAMA Network Open*.

	Primary Organ Fail Reason	Primary reason for organ failure from the United Network for Organ Sharing
**MAR**		Medications administered between transplant admit date and admin censor date
	Study ID	Deidentified ID representing one referral, 13 digits
	Patient ID	Deidentified ID representing one patient, first 11 digits of the Study ID
	Encounter ID	Deidentified ID representing a unique patient contact
	Linked Encounter	Y if this encounter is linked to the transplant referral
	Order Medication ID	Deidentified ID representing a unique order for the patient on this date
	Line	Line of the current medication order (represents an administration)
	Days from Referral to Order	Number of days from Referral Date to Order Date
	Days from Referral to Order Start	Number of days from Referral Date to Order Start Date
	Days from Referral to Order End	Number of days from Referral Date to Order End Date
	Days from Referral to Schedule	Number of days from Referral Date to Scheduled Date
	Days from Referral to Taken	Number of days from Referral Date to Taken Time
	MAR Action	Action category for this medicine (e.g. Given, Missed)
	MAR Action Reason	Reason that is given for documenting a certain action (e.g. Patient/Family refused)
	Medication Name	Name of Medication
	Medication Simple Generic	Generic name listed for each medication
	Pharmacy Class	Pharmaceutical class indicating the chemical families the drug belongs to
	Pharmacy Subclass	First pharmaceutical subclass
	Route	Route the drug was administered
	Dose	Dose value of the administration
	Dose Unit	Unit of the dose
	Strength	Strength of this NDC version of the drug
	Infusion Rate	Rate at which the medication was infused
	Infusion Rate Unit	Unit of the infused medication
**Blood Admin**		Blood Administration for all encounters
	Study ID	Deidentified ID representing one referral, 13 digits
	Patient ID	Deidentified ID representing one patient, first 11 digits of the Study ID
	Encounter ID	Deidentified ID representing a unique patient contact

© 2025 McElroy LM et al. *JAMA Network Open*.

	Linked Encounter	Y if this encounter is linked to the transplant referral, N if the encounter is not linked
	Days from Referral to Blood Order	Number of days from Referral Date to Order Date
	Days from Referral to Blood Admin	Number of days from Referral Date to Blood Admin
	Blood Product Category	Category of blood that was administered. We are using 4 categories: FFP (Fresh Frozen Plasma), RBC (Red Blood Cells), Platelets, Cryo (precipitate)
	Total Units per Day	Sum of the units per type, per day
**Immunization**		Immunization Records
	Study ID	Deidentified ID representing one referral, 13 digits
	Patient ID	Deidentified ID representing one patient, first 11 digits of the Study ID
	Encounter ID	Deidentified ID representing a unique patient contact
	Days from Referral to Immunization	Days from Referral to immunization date
	Days from Referral to Expiration	Days from Referral to expiration date
	Immunization Status	Status of the immunization (e.g. Given, Deferred)
	IMM Product	Item which stores the product of the immunization. Products are usually related to the lot number.
	Immunization Name	Name of the immunization
	External Admin Type	Source of verification for external administration of immunization
	External Admin Flag	Indicates if the immunization was administered locally or historically and reported by another source
**Measure**		**Measurements: 6 minute walk test, Karnofsky Score, Waist to Hip Ratio, ECMO, CRRT, Trach, ETT, Ejection Fraction, Right Ventricular Systolic Pressure**
	Study ID	Deidentified ID representing one referral, 13 digits
	Patient ID	Deidentified ID representing one patient, first 11 digits of the Study ID
	Days from Referral to Measure	Number of days from Referral Date to measurement recorded date
	Measure Time	Time of measurement; collected for those measurements that are time sensitive
	Measure Name	Name of measurement
	Measure Display Name	Display name of measurement
	Measure Value	Value of the measure
	Measure Group	Grouping of this measurement if measure has more than one field that needs to be pulled in
**Cost**		All patient cost data beginning one year prior to referral
	Study ID	Deidentified ID representing one referral, 13 digits

© 2025 McElroy LM et al. *JAMA Network Open*.

	Patient ID	Deidentified ID representing one patient, first 11 digits of the Study ID
	Days from Referral to Cost Begin	Number of days from Referral to Admit Date on hospital account
	Days from Referral to Cost End	Number of days from Referral to Discharge Date on hospital account
	Payments	Total payments posted to an account, self-pay or insurance
	Patient Payment Amount	Amount of the payments on the account that were made by the patient and not insurance
	Patient Responsibility	Amount of charges assigned to the patient
	Bad Debt Amount	Amount of AR on the account that is classified as Bad Debt
**Address History**		Address key held by sites locally in a secure location; it contains information used as input to the Degauss SDOH container to generate area level SDOH via pipeline
	Study ID	Deidentified ID representing one referral, 13 digits
	Patient ID	Deidentified ID representing one patient, first 11 digits of the Study ID
	Row Number	Row number of address in historical order with most recent as row one
	Days from Referral to Address Effective Date	Days from Referral to the date this address was effective for this patient
	Days from Referral to Address End Date	Days from Referral to the date this address is no longer used or null if this is the current
	Street Address	Line 1 of the patient's street address
	City	The Patient's city
	State	The Patient's State
	Zip	5 digit zip code
	Zip4	4 plus digit zip, if available
	County	The patient's county
**SDOH**		Area-level Social Determinants of Health (generated via McElroy SDOH pipeline)
	Study ID	Deidentified ID representing one referral, 13 digits
	Patient ID	Deidentified ID representing one patient, first 11 digits of the Study ID
	Row Number	Row number of address in historical order with most recent as row one
	Days from Referral to Address Effective Date	Days from Referral to the date this address was effective for this patient
	Days from Referral to Address End Date	Days from Referral to the date this address is no longer used or null if this is the current
	Minimum Wage	State minimum wage
	Per Union Member	Percentage of total employed who have union membership

© 2025 McElroy LM et al. *JAMA Network Open*.

	Per Union Rep	Percentage of total employed who are represented by a union
	PCP	Primary Care Physician, Patient Care Non-Fed 2018
	Psychiatry	Psychiatry, Total Patient Care Non-Fed 2018
	Dentist	Dentists with NPI 2018
	Social Work	Hosp with Social Work Services Short Term General Hospitals 2018
	Mental Health	Community Mental Health Centers 2018
	Total Population	Total county population (juvenile court records)
	Pop 10 Through Upper Age	County population from age 10 through upper age (juvenile court records)
	Pop 0 Through Upper Age	County population from age 0 through upper age (juvenile court records)
	Delinquency Petition	Number of juvenile court cases with delinquency petition status
	Delinquency Non-Petition	Number of juvenile court cases with delinquency non-petition status
	Status Petition	Number of juvenile court cases with status non-petition status
	Status Non-Petition	Number of juvenile court cases with status non-petition status
	RFEI	Food swamp index
	RPL Themes	SVI
	ACS B09005_001	Population under 18 years in households, total
	ACS B09005_004	Population under 18 years in households, in male householder, no spouse/partner present household
	ACS B09005_005	Population under 18 years in households, In female householder, no spouse/partner present household
	ACS B19083_001	Gini index of income inequality in all households
	ACS B09010_001	Population under 18 years in households, total
	ACS B09010_002	Living in household with supplemental Security Income (SSI), cash public assistance income, or Food Stamps/SNAP in past 12 months, under 18 years in household
	ACS B25106_001	Housing costs as percentage of household income in past 12 months, total occupied housing units
	ACS B25106_006	Housing costs as percentage of household income in past 12 months, owner-occupied, income less than $20,000, 30 percent or more
	ACS B25106_010	Housing costs as percentage of household income in past 12 months, owner-occupied, income $20,000 to $34,999, 30 percent or more
	ACS B25106_014	Housing costs as percentage of household income in past 12 months, owner-occupied, income $35,000 to $49,000, 30 percent or more
	ACS B25106_018	Housing costs as percentage of household income in past 12 months, owner-occupied, income $50,000 to $74,000, 30 percent or more

© 2025 McElroy LM et al. *JAMA Network Open*.

	ACS B25106_022	Housing costs as percentage of household income in past 12 months, owner-occupied, income 75,000 or more, 30 percent or more
	ACS B25106_028	Housing costs as percentage of household income in past 12 months, renter-occupied, income less than $20,000, 30 percent or more
	ACS B25106_032	Housing costs as percentage of household income in past 12 months, renter-occupied, income $20,000 to $34,999, 30 percent or more
	ACS B25106_036	Housing costs as percentage of household income in past 12 months, renter-occupied, income $35,000 to $49,000, 30 percent or more
	ACS B25106_040	Housing costs as percentage of household income in past 12 months, renter-occupied, income $50,000 to $74,000, 30 percent or more
	ACS B25106_044	Housing costs as percentage of household income in past 12 months, renter-occupied, income 75,000 or more, 30 percent or more
	ACS S0802_C01_081	Travel time to work for workers 16 years and over who did not work from home, less than 10 min
	ACS S0802_C01_082	Travel time to work for workers 16 years and over who did not work from home, 10 to 14 min
	ACS S0802_C01_083	Travel time to work for workers 16 years and over who did not work from home, 15 to 19 min
	ACS S0802_C01_084	Travel time to work for workers 16 years and over who did not work from home, 20 to 24 min
	ACS S0802_C01_085	Travel time to work for workers 16 years and over who did not work from home, 25 to 29 min
	ACS S0802_C01_086	Travel time to work for workers 16 years and over who did not work from home, 30 to 34 min
	ACS S0802_C01_087	Travel time to work for workers 16 years and over who did not work from home, 35 to 44 min
	ACS S0802_C01_088	Travel time to work for workers 16 years and over who did not work from home, 45 to 59 min
	ACS S0802_C01_089	Travel time to work for workers 16 years and over who did not work from home, 60 or more min
	ACS DP04_0046P	Homeownership, percentage of owner-occupied housing units
	Health literacy estimate	Health literacy estimate
	ADI_NATRANK	National ADI percentile (2015)
	ADI_STATERNK	State ADI percentile (2015)
	D_LDPNT_2	EJ Index for % pre-1960 housing (lead paint indicator)
	D_DSLPM_2	EJ Index for Diesel particulate matter level in air
	D_CANCR_2	EJ Index for Air toxics cancer risk
	D_RESP_2	EJ Index for Air toxics respiratory hazard index

© 2025 McElroy LM et al. *JAMA Network Open*.

	D_PTRAF_2	EJ Index for Traffic proximity and volume
	D_PWDIS_2	EJ Index for Indicator for major direct dischargers to water
	D_PNPL_2	EJ Index for Proximity to National Priorities List (NPL) sites
	D_PRMP_2	EJ Index for Proximity to Risk Management Plan (RMP) facilities
	D_PTSDF_2	EJ Index for Proximity to Treatment Storage and Disposal (TSDF) facilities
	D_OZONE_2	EJ Index for Ozone level in air
	D_PM25_2	EJ Index for PM2.5 level in air
	ACS B27010_001	Health insurance, total civilian non-institutionalized population
	ACS B27010_017	Health insurance, no health insurance coverage, under 19 years
	ACS B27010_033	Health insurance, no health insurance coverage, 19 to 34 years
	ACS B27010_050	Health insurance, no health insurance coverage, 35 to 64 years
	ACS B27010_066	Health insurance, no health insurance coverage, 65 years and older
	ACS B15002_001	Education attainment, total population 25 years and over
	ACS B15002_011	Education attainment, high school, 25 years and over, male
	ACS B15002_028	Education attainment, high school, 25 years and over, female
	ACS B15002_015	Education attainment, bachelor's degree, 25 years and over, male
	ACS B15002_032	Education attainment, bachelor's degree, 25 years and over, female
	ACS B19001_001	Household income, total households
	ACS B19001_002	Household income, households less than $10,000
	ACS B19001_003	Household income, households $10,000 to $14,999
	ACS B19001_004	Household income, households $15,000 to $19,999
	ACS B19001_015	Household income, households $125,000 to $149,999
	ACS B19001_016	Household income, households $150,000 to $199,999
	ACS B19001_017	Household income, households $200,000 or more
	ACS B28011_001	Internet subscriptions in household, total households
	ACS B28011_004	Internet subscriptions in household, households with broadband such as cable, fiber optic, or DSL

Accounts Receivable; ADI: Area Deprivation Index; BMI: Body Mass index; DEA: Drug Enforcement Agency; ED: Emergency Department; EJ: Environmental Justice; FFP: fresh frozen plasma; ICD: International Classification of Diseases; ICU: Intensive Care Unit; ID: identification; MELD: Model for End Stage Liver Disease; N: No; RBC: Red blood cell; UNOS: United Network for Organ Sharing; Y: Yes

© 2025 McElroy LM et al. *JAMA Network Open*.
