# Supplementary material for: Development of Common Data Elements for Organ Transplantation
Source: JAMA Netw Open. 2025 Apr 28;8(4):e257704. doi: 10.1001/jamanetworkopen.2025.7704 (PMC12038510; doi:10.1001/jamanetworkopen.2025.7704)
Supplement: Supplement 2. — Data Sharing Statement [file jamanetwopen-e257704-s002.pdf]

Data Sharing Statement

Data
Data available: Yes
Data types: Data dictionary
How to access data: provided as SDC
When available: With publication

Supporting Documents
Document types: None

Additional Information
Who can access the data: the data element list was provided with the manuscript
Types of analyses: none
Mechanisms of data availability: see above
Any additional restrictions: none
